# Thumb Schwannoma : A Frequent Misdiagnosis

**DOI:** 10.5704/MOJ.1603.010

**Published:** 2016-03

**Authors:** SS Mooi, TS Ahmad

**Affiliations:** Department of Orthopaedics, University of Malaya, Kuala Lumpur, Malaysia

**Keywords:** Thumb schwannoma

## Abstract

A 32 year-old Malay lady presented with a swelling over the dorsal surface of her right thumb for 6 months. The swelling was non-tender, smooth surfaced, mobile and nonfluctuating with no bony involvement. The provisional diagnosis was ganglion cyst. Excisional biopsy did not show features of ganglion cyst as it appeared to be wellencapsulated, multi-lobulated and yellowish in colour. Histopathological examination showed that it was a schwannoma. Schwannomas are relatively rare benign tumours which are frequently misdiagnosed. In this case, it was misdiagnosed both as a ganglion and a lipoma.

## Case Report

A 32 year-old healthy lady presented with a swelling over the dorsal surface of her right thumb for a duration of six months. The swelling increased in size gradually over the last two months with no pain and no neurological symptoms. She did not experience constitutional symptoms. The presenting complaint was due to the unsightly swelling over her right thumb.

Clinically, there was a 2cm X 2cm smooth surfaced, non-tender, mobile, multilobulated firm swelling over the dorsal surface of the proximal phalanx of her right thumb. The swelling was not pulsatile and did not shrink on elevation of her right upper limb. The range of motion of the thumb was full passively and actively. The sensation of the affected thumb was intact. Radiologically, there was no bony involvement. A provisional diagnosis of ganglion cyst of the right thumb was made.

**Fig. 1:**
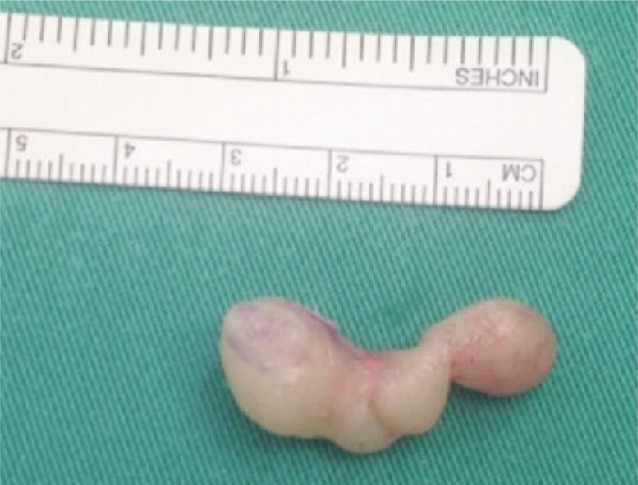
Excisional biopsy revealed a multi-lobulated, well-encapsulated, yellowish tumour.

An excision biopsy under general anaesthesia revealed a multi-lobulated, well-capsulated and yellowish swelling (Fig 1). We diagnosed it as a lipoma with a differential diagnosis of schwannoma.

Our patient recovered uneventfully. The histopathology report revealed an encapsulated solid tumour composed of irregularly arranged spindle cells in a fibrous background with hypercellular (Antoni A) and focal hypocellular (Antoni B) areas seen. Verocay bodies were present. The spindle cells were cytologically benign with fairly monomorphic bland nuclei, inconspicuous nucleoli and immunoreactive for S-100. Mitoses and necrosis were not seen. The histopathological interpretation was schwannoma.

## Discussions

Strickland and colleague (1977) reported that primary neural tumours of the hand are rare and represent less than 5% of soft-tissue neoplasms of the upper extremities^1^. Ozdemir (2005) described that schwannomas are the most common primary solitary tumours among peripheral nerve tumours. Schwannomas originate from the cells of the Schwann sheath and are surrounded by epineurium, thus the name schwannoma. The usual nature of schwannoma is that it usually grows slowly without pain. Due to its painless nature, the first presentation can be very late following onset of the swelling. They usually occur in patients aged 30 to 60 years and there is no race or sex predilection. Schwannomas are usually solitary. The treatment of choice is extracapsular or intracapsular removal under magnification^2^.

Lincoski *et al*(2007) mentioned schwannomas are well encapsulated, and since the nerve fibers typically do not enter the tumour, complete excision of schwannoma is usually possible^3^. The authors also highlighted that the diagnosis is not often made before surgery. Schwannomas are frequently being diagnosed as ganglia, giant cell tumours and lipomata. Miles *et al* (2010) also reported that schwannomas are frequently diagnosed as ganglion cysts^4^. The authors above described that schwannomas have a low recurrence rate following complete excision^1-4^.

The authors diagnosed as a ganglion cyst without other radiological investigations such as ultrasonography or MRI due to the relative rarity of schwannoma in the hand or finger.

We are currently doing consultation room ultrasonography to aid the diagnosis of hand swellings to minimise unnecessary misdiagnosis of schwannomas and other benign and malignant tumours of hand.
